# An update on cannabis products, cannabinoid content, plant type, and prices five years after legalization in Ontario, Canada

**DOI:** 10.1186/s42238-026-00444-6

**Published:** 2026-05-07

**Authors:** Paulina Antwi, Justin Matheson, Lohith Balaraju, Christine M. Wickens, Bernard Le Foll, Sergio Rueda, Patricia Di Ciano

**Affiliations:** 1 Centre for Addiction and Mental Health, Institute for Mental Health Policy Research, 250 College Street, Toronto, ON M5T 1R8 Canada; 2 Campbell Family Mental Health Research Institute, Centre for Addiction and Mental Health, Toronto, ON Canada; 3 Department of Pharmacology and Toxicology, Temerty Faculty of Medicine, University of Toronto, Toronto, ON Canada; 4 Dalla Lana School of Public Health, University of Toronto, Toronto, ON Canada; 5 Institute of Health Policy, Management and Evaluation, University of Toronto, Toronto, ON Canada; 6 Addictions Division, Centre for Addiction and Mental Health, Toronto, ON Canada; 7 Institute of Medical Science, Temerty Faculty of Medicine, University of Toronto, Toronto, ON Canada; 8 Department of Psychiatry, Temerty Faculty of Medicine, University of Toronto, Toronto, ON Canada; 9 Department of Family and Community Medicine, University of Toronto, Toronto, ON Canada; 10 Waypoint Research Institute, Waypoint Centre for Mental Health Care, Penetanguishene, ON Canada

**Keywords:** THC, CBD, Cannabis, Legalization, Retail market, Potency

## Abstract

**Introduction:**

Changing trends in legal recreational cannabis markets necessitates the continuous monitoring of important market metrics such as price, potency, type, and availability of cannabis products which may influence consumer purchasing behavior. After initial legalization of recreational cannabis use in Canada in 2018 and expansion of the legal market in 2019, an increase in product types and decrease in prices were observed. The purpose of the present study was to provide an update on products on offer to cannabis consumers in Ontario, the largest Cannabis market in Canada, five years after initial federal legalization of non-medical cannabis use.

**Method:**

Data were obtained from the Ontario Cannabis Store (OCS) Website, the sole wholesaler and online market in Ontario between January and April 2024. Information on product type, prices, delta-9-tetrahydrocannabinol (THC) and cannabidiol (CBD) potency and plant type was extracted and products were grouped by OCS categories and sub-categories, and by route of administration (Inhaled, ingestible, topical and others).

**Results:**

A total of 1727 products were mapped, of which the majority available were inhaled products, sub-divided into smoking, vaping or concentrates (n = 1271). The majority of inhaled products were predominantly indica-dominant while most ingestible products (e.g. edibles) were mainly sativa-dominant. Most inhaled products were “very strong products”, i.e., THC potency of 20% or greater (greater than 97%, 96% and 99% of all dried flower, vaping and concentrate products, respectively). All edibles were within the legally acceptable limit of 10 mg of THC per container. Average prices were lowest for dried flower products ($7.91/g) among inhaled products (pre-rolls: $9.65/g, 510 thread cartridges: $37.7/g, infused flower: $18.66/g). Compared to our previous study in 2022, which utilized data from the same website and similar extraction methods, the total number of products on the legal market declined, while the number of edible products (particularly gummies) and disposable vapes, increased. Prices of all flower products (*p* < 0.001), most abundant vapes (disposable vapes and thread cartridges: *p* < 0.001) and soft chews (*p* < 0.027) decreased in 2024 compared to 2022 at the 5% significance level. Conversely, THC potencies of products available generally increased (*p* < 0.05).

**Discussion:**

Given the trend of decreasing price and changes in products available, continuous monitoring of the cannabis market is vital. This is necessary to assess the consequences of legalization and inform legal amendments such as price control and potency limits that may curb potential negative consequences possible with the current market trend.

## Introduction

The legalization of non-medical cannabis in Canada has been associated with changes in market trends over time, including changes in product type, product preferences and price (Matheson and Le Foll 2020; Tassone et al. 2023). Consumer purchasing behavior has been shown to be impacted by price, potency of delta-9-tetrahydrocannabinol (THC; the psychoactive component of cannabis) and availability of cannabis products in legal and non-legal markets (Freeman et al. 2019; Okey et al. 2025; Donnan et al. 2024; Donnan et al. 2023). In the state of Washington, in the United States (US), the cannabis market saw an increase in high-THC products after legalization and most flower products had THC concentrations greater than 20%; THC concentrates of most extracts were greater than 60%. The market share of inhalable extracts experienced a 145.8% increase after legalization in 2012 (Smart et al. 2017). Similarly, a proliferation in the number of cannabis products available and significantly decreasing prices of cannabis products in legalized regions such as Colorado and Oregon have also been observed (Kilmer and Perez-Davila 2023; Murray and Hall 2020; Firth et al. 2020).

In Canada, the cannabis market continues to rapidly evolve post-legalization. Canada legalized dried flower cannabis and cannabis oil production, distribution, sale and possession in October 2018 (Cannabis Act 2018), becoming the second country worldwide after Uruguay to legalize personal use of cannabis for non-medical purposes. Edibles, extracts, and topical cannabis legalization followed the next year under the Cannabis Act (Government of Canada 2019). The federal government highlighted certain aims of legalization, including protecting the health of young people by restricting access to cannabis through regulation, “protect public health and safety by allowing adults access to legal cannabis”, and providing a quality-controlled cannabis supply (Hammond et al. 2022; Hall and Lynskey 2020; Rubin-Kahana 2022 p.2).

Despite these intentions, a primary public health concern has been a potential increase in the prevalence and magnitude of cannabis use and related harms that may result from widespread access to the retail cannabis market and expanded product types and potencies associated with legalization (Myran et al. 2023; Center for Addiction and Mental Health 2014). The corresponding implications of legalization reported in Canada so far have included increased use among adults (Di Ciano et al. 2025; Fischer et al. 2023; McDonald et al. 2025), increased incidence of emergency department visits and hospitalizations related to cannabis use (Fischer et al. 2023; McDonald et al. 2025), mixed effects among adolescent use and drivers under the influence of cannabis (2022; Fischer et al. 2023; Imtiaz et al. 2024), a decrease in arrests and convictions, and a decline in both the illegal and medical cannabis markets (Fischer et al. 2023; Dion et al. 2024; Callaghan et al. 2024). The flourishing legal cannabis market has also afforded the government a significant tax-revenue stream (McDonald et al. 2025).

According to the 2024 Annual Ontario Cannabis market report, there were 1726 authorized cannabis stores in Ontario, the most populous province in Canada (Ontario Cannabis Store 2024). As of August 2025, this number was up to 1809 authorized cannabis stores in Ontario, and 33 stores pending authorization (Alcohol and Gaming Commission of Ontario 2025). In the market, preference among consumers for processed products such as concentrates and edibles has increased in Canada (Tassone et al. 2023; McDonald et al. 2025; Hammond et al. 2022). Increased access to various cannabis products and a decline in prices, attributed to competition from the regulated market have been observed post-legalization in the Canadian province of British Columbia (Dodd et al. 2025). Our previous study, which described product offerings 3 years post-legalization in the largest cannabis market in Canada (the province of Ontario), found that a wide array of cannabis product types and formulations were available to meet consumer preferences with an overall decrease in prices compared to the pre-legalization era (Tassone et al. 2023). Alternative cannabis products such as edibles, which are of concern due to their late and unpredictable onset and long duration of action, may contribute to increasing rates of accidental ingestions and emergency room visits as observed in Colorado (Matheson and Le Foll 2020). Declining prices may contribute to an increase in cannabis use as evidenced by lessons from tobacco and alcohol legalizations, which indicate the inverse relationship between price and consumption (Budney and Borodovsky 2017; Wagenaar et al. 2009).

We also observed variations in the potency of THC (high vs low vs mixed THC-CBD ratios) and a strong market for high-THC products (Tassone et al. 2023). Studies have shown that the consumption of high-THC potency products have both acute and long-term detrimental effects on health and have a potentially higher risk of harm (Freeman et al. 2019; Pennypacker et al. 2022). High potency cannabis has been found to be associated with increased dependence severity, especially in young individuals (Freeman and Winstock 2015), psychosis risk and cannabis use disorder (Petrilli et al. 2022; Lake et al. 2025). The increased availability of high-potency products may also be associated with more desirable rewarding effects (dose-dependent reinforcement effects) leading to increased likelihood of repeated use, particularly for first-time users (Budney and Borodovsky 2017).

The impact of factors such as cannabis accessibility, product type availability, and price on consumer behavior, and availability of higher THC products in the market, may therefore have serious public health consequences. The continued evaluation of the Canadian cannabis market, including assessment of cannabis product diversity, is therefore critical in protecting public health and safety, a key public health objective of the Canadian government pre-legalization, and in assessing the impact of cannabis legalization (Rubin-Kahana 2022; Goodman et al. 2020). The aim of this study is to provide an update on the legalized cannabis market in Ontario, five years after initial federal legalization of non-medical cannabis use. We sought to evaluate products available on the market, their prices, and potencies of THC and cannabidiol (CBD), using data from the Ontario Cannabis Store (OCS), the only wholesale and online retailer in Ontario. We provide an update to our previous study in 2022 (Tassone et al. 2023) assessing similar metrics and compare findings from both studies, which may be insightful in determining how quickly market trends change. Cannabis market trend insights could inform further legislative amendments and policy- making towards minimizing cannabis- related harms and safeguarding public health.

## Method

This cross-sectional study was designed to assess the Ontario cannabis market in the first quarter of 2024; five years post-legalization. Similar to our previous study (Tassone et al. 2023), data were extracted from the OCS website to obtain information on product types, plant type, THC and CBD potency, and price of products. The OCS is the sole wholesale and online retailer and hence the only distributor of legal cannabis products in Ontario. Products outlined are therefore the same products found and sold across all retail cannabis stores in Ontario. Our study only captures retail products and prices provided on the OCS website from January to April, 2024. Product information was inputted manually and analyzed on Microsoft Excel and missing data were labelled as N/A and excluded.

All data were analyzed using descriptive statistic measures: means (M), standard deviations (SD), and ranges. Pivot tables were used to aggregate select variables to determine averages, product counts, and sums. We also categorized OCS categories and sub-categories by route and method of administration to provide a better understanding of the ways various cannabis products are used.

Unlike consumer purchased individual product labels which indicate a single THC or CBD value for products, the OCS website lists products with THC and CBD contents indicated as a range of values (in %/g or mg/unit) (Ontario Cannabis Store 2025). For ingestible products, regulations placed THC limits per package. Therefore, the average for each product’s THC and CBD range was calculated and then averaged by its sub-category. The OCS also labels products containing 20% or greater THC as “very strong THC” levels and we use this as a threshold for THC potency (Ontario Cannabis Store 2022). We computed the frequency and proportion of such products using the unit% of each product. The OCS does not define “very strong CBD” levels; hence, no such classification is included. We present a percentage of products greater than a threshold of 5% (an empirical benchmark) only to provide information on CBD potencies available on the market. There are no universally accepted benchmarks for CBD potency classification. Using pivot tables, alphabetical sorting and manual counting, we calculated the frequency of very strong THC and ≥ 5% CBD products by product sub-category. Products were categorized by plant type into hybrid, indica-dominant, or sativa-dominant by sorting alphabetically and counting all products manually for each product sub-category.

Due to the product variations within each group, utilizing metrics such as average price can be challenging and as such, a feasible approach is to compute prices per sub-group (Dilley et al. 2023). Individual products listed often had multiple selling prices depending on the quantity available for purchase. As such, for each product, we computed the average of the highest and lowest selling price and then computed the average price per sub-category (in $/g, $/0.1 g, $/ml, or $/unit). We also calculated the minimum and maximum selling price per sub-category, thereby allowing us to estimate the approximate cost of a bulk purchase of a particular product by a consumer. Prices for products in the inhaled-vaping category were converted to $/g from $/0.1 g units reported on the OCS website to facilitate meaningful comparisons. Original units were retained for all other products.

Most dried flower products were only available in one or two quantities while pre-rolls were available in varying quantities, making reporting average prices, as observed in previous studies, challenging. As such, the lowest and highest price per gram of each product was determined to provide clarity.

We conducted independent samples t-tests as the data sets were largely independent to analyze and compare the prices and THC concentrations of selected product sub-categories in 2024 to the previous findings in 2022, which utilized the same data source (OCS website) and extraction methods. Selected product categories were consistent across both years and/or most abundant product types. The independent variable was year (2022 versus 2024) while dependent variables were the prices and THC and CBD concentrations. Normality and homogeneity of variance were analyzed using the Levene’s test and significance level was set at *p* < 0.05. IBM SPSS Statistics, Version 27, was used for this analysis.

## Results

### Product categories and routes of administration

A total of 1727 products categorized into 7 main product types, each with subcategories: flower- dried flower, pre-rolls and seeds (n = 728), vapes- disposable vapes, 510 thread cartridges, pax pods and closed loop pods (n = 296), concentrates- infused flower, hash, shatter, rosin, wax, kief and resin (n = 264), edibles- chocolates, baked goods, gummies, hard edibles, savory snacks and pantry (n = 188), beverages- coffees & teas, soft drinks, sparkling waters, beverage mixers, juices and dealcoholized (n = 93), extracts- oils (drops and sprays), capsules and sublingual strips (n = 104), and topicals- creams & lotions, transdermal gels, intimacy oils and bath & shower (n = 54) were extracted. These were condensed into four main categories based on the route of administration. Flower (except seeds), vapes and concentrates were classified as inhaled products; edibles, beverages, oil drops and sprays, capsules and sublingual strips as ingestible; topicals, made up of creams and lotions, transdermal gels and intimacy oils and others which included seeds, pantry and baths & showers. Products that may be used via more than one route are classified in more than one category. Of the four categories, inhaled products made up the majority (n = 1271). Within each category, sub-categories with the most products included: dried flower (n = 373), 510 thread cartridges (n = 224), infused flower (n = 143), gummies (n = 130), beverages (n = 93), oils- drops (n = 40), capsules (n = 49) sublingual strips (n = 4), topicals (n = 41) and seeds (n = 17). (See Tables 1 and 2).

**Table 1:** Ontario cannabis store (OCS) product categories and sub-categories mapped by route of administration (RoA)

OCS categories	OCS sub-categories	RoA categories	ROA sub-categories
Flower	Dried Flower	Inhaled—smoking	Dried Flower
	Pre-Rolls		Pre-Rolls
	Seeds		
Vapes	Disposable Vapes	Inhaled—vaping	Dried Flower
			Disposable Vapes
	510 Thread Cartridges^a^		510 Thread Cartridges
	Pax Pods^b^		Pax Pods
	Closed Loop Pods^c^		Closed Loop Pods
Concentrates	Infused Flower		Infused Flower
	Hash		Hash
	Shatter		Shatter
	Rosin	Inhaled—concentrates	Rosin
	Wax		Wax
	Kief		Kief
	Resin		Resin
Edibles	Chocolates	Ingestible—Edibles	Chocolates
	Baked Goods		Baked Goods
	Gummies		Gummies
	Hard Edibles		Hard Edibles
	Savory Snacks		Savory Snacks
	Pantry^d^		Pantry^d^
Extracts	Oils- Drops	Ingestible—Oils	Drops
	Oils- Spray		Spray
	Capsules	Ingestible—Capsules	Capsules
	Sublingual Strips	Ingestible—Sublingual Strips	Sublingual Strips
Beverages	Coffees & Teas	Ingestible—Beverages	Beverages
	Soft Drinks		
	Sparkling Waters		
	Beverage Mixers		
	Juices		
	Dealcoholized		
Topicals	Creams & Lotions	Topicals	Topicals
	Transdermal Gels		
	Intimacy Oils		
	Bath & Shower	Others	Bath & Shower
			Seeds

^a^Vaping products compatible with standard 510 batteries

^b^Vaping products designed exclusively for the PAX Era vaporizer

^c^Brand-specific vaping systems where the device and pods are exclusive to a single manufacturer.”

^d^Cannabis infused edible cooking and baking ingredients and staples

**Table 2:** Tetrahydrocannabinol (THC) and cannabidiol (CBD) potency by product type (first quarter of 2024)

Category	No. of Products	M *(SD)* [Range]		No. of products (%) above specified threshold	
		**THC**	**CBD**	**THC (≥ 20%/g)**	**CBD (≥ 5%/g)**
Inhaled—smoking (%/g)					
Dried flower	373	26 *(4)* [0—40]	1 *(3)* [0—20]	361 (97)	79 (21)
Pre-rolls	338	25 *(5)* [0—38]	1 *(3)* [0—22]	281 (83)	66 (20)
Inhaled—vaping (%/g)					
Disposable Vapes	56	82 *(15)* [0—96]	4 *(13)* [0—76]	54 (96)	22 (39)
510 Thread Cartridges^a^	224	82 *(12)* [0—96]	3 *(8)* [0—57]	223 (99)	82 (37)
Pax Pods^b^	8	84 *(2)* [80—93]	1 *(0)* [0—1]	8 (100)	0 (0)
Closed Loop Pods^c^	8	86 *(0)* [83—89]	1 *(0)* [0—2]	8 (100)	0 (0)
Inhaled—Concentrates (%/g)					
Infused Flower	143	38 *(5)* [9-56]	2 *(3)* [0—31]	142 (99)	24 (17)
Hash	44	44 *(8)* [23-68]	2 *(4)* [0—26]	44 (100)	11 (25)
Shatter	21	67 *(19)* [28—86]	2 *(1)* [0—8]	21 (100)	9 (43)
Rosin	18	54 *(18)* [30—85]	5 *(12)* [0—55]	18 (100)	7 (39)
Wax	3	54 *(20)* [38—81]	2 *(1)* [0—6]	3 (100)	1 (33)
Kief & Sift	6	36 *(7) *[25-50]	1 *(0)* [0—4]	6 (100)	0 (0)
Resin	29	80 *(8)* [62—96]	2 *(2)* [0—20]	29 (100)	10 (34)
Ingestible – Edibles (mg/unit, mg)					
Chocolates	20	10 *(0)* [8-10]	3 *(4)* [0—10]	N/A	N/A
Baked Goods	20	9 *(2)* [0—10]	4 *(11)* [0—50]	N/A	N/A
Gummies	130	9 *(2)* [0—10]	42 *(144)* [0—900]	N/A	N/A
Hard Edibles	2	9 *(1)* [8-10]	6 *(6)* [0—10]	N/A	N/A
Savory Snacks	8	9 *(1)* [8-10]	4 *(7)* [0—20]	N/A	N/A
Pantry (mg)^d^	8	9 *(2)* [3-10]	19 *(37)* [0—115]	N/A	N/A
Ingestible – Beverages (mg/unit, mg)					
Beverages	93	8 *(3)* [0—10]	8 *(19)* [0—100]	N/A	N/A
Ingestible – Oils (mg/g, mg/spray)					
Oil-Drops	40	13 *(47)* [0—310]	22 *(40)* [0—210]	0 (0)	0 (0)
Oils-Spray	11	3 *(4)* [0—10]	4 *(9)* [0—25]	0 (0)	0 (0)
Ingestible – Capsules (mg/unit, mg)					
Capsules	49	5 *(4)* [0—12]	19 *(27)* [0—105]	N/A	N/A
Ingestible—Sublingual Strips (mg/unit, mg)					
Sublingual Strips	4	9 *(1)*[9-12]	0—1 *(1)* [0—1]	N/A	N/A
Topicals (mg)					
Topicals	41	155 *(254)* [0—100]	636 *(655)* [0—2100]	N/A	N/A
Others					
Seeds	17	N/A	N/A	N/A	N/A
Bath & Shower (mg)	13	37 *(82)* [0—345]	277 *(243)* [43—1150]	N/A	N/A

^a^Vaping products compatible with standard 510 batteries

^b^Vaping products designed exclusively for the PAX Era vaporizer

^c^Brand-specific vaping systems where the device and pods are exclusive to a single manufacturer.”

^d^Cannabis infused edible cooking and baking ingredients and staples

### Plant type

Information on listed plant types- blend, hybrid, indica-dominant, and sativa-dominant- was provided for all products (see Table 3**)**. The majority of inhaled products had a higher percentage of indica-dominant versus sativa-dominant products (dried flower (42% vs 27%), pre-rolls (36% vs 29%), infused flower (32% vs 25%), hash (27% vs 16%), shatter (48% vs 19%), rosin (61% vs 5%), kief & sift (17% vs 0%), and resin (38% vs 24%)). Conversely, vape products were predominantly sativa-dominant (disposable vapes (21% vs 36%), 510 thread cartridges (22% vs 32%), and pax pods (25% vs 50%)). Almost all ingestible products (edibles, beverages, oils, and capsules) tended to be a blend. Similarly, 41% of topicals were a blend while 75% of sublingual strips were hybrid.

**Table 3:** Plant type by product type (First Quarter of 2024)

Category	No. of products				
	**Total**	**Blend**	**Hybrid**	**Indica-dominant**	**Sativa-dominant**
Inhaled-smoking					
Dried flower	373	5 (1%)	109 (29%)	158 (42%)	101 (27%)
Pre-rolls	338	20 (6%)	97 (29%)	122 (36%)	99 (29%)
Inhaled-vaping					
Disposable Vapes	56	3 (5%)	21 (38%)	12 (21%)	20 (36%)
510 Thread Cartridges^a^	224	42 (19%)	61 (27%)	50 (22%)	71 (32%)
Pax Pods^b^	8	1 (13%)	1 (13%)	2 (25%)	4 (50%)
Closed Loop Pods^c^	8	2 (25%)	2 (25%)	2 (25%)	2 (25%)
Inhaled—Concentrates					
Infused Flower	143	21 (15%)	40 (28%)	46 (32%)	36 (25%)
Hash	44	15 (34%)	10 (23%)	12 (27%)	7 (16%)
Shatter	21	2 (10%)	5 (24%)	10 (48%)	4 (19%)
Rosin	18	0 (0%)	6 (34%)	11 (61%)	1 (5%)
Wax	3	0 (0%)	1 (33%)	1 (33%)	1 (33%)
Kief & Sift	6	1 (17%)	4 (67%)	1 (17%)	0 (0%)
Resin	29	2 (7%)	9 (31%)	11 (38%)	7 (24%)
Ingestible—Edibles					
Chocolates	20	16 (80%)	4 (20%)	0 (0%)	0 (0%)
Baked Goods	20	6 (30%)	5 (25%)	2 (10)	7 (35%)
Gummies	130	28 (22%)	41 (32%)	33 (25%)	28 (22%)
Hard Edibles	2	2 (100%)	0 (0%)	0 (0%)	0 (0%)
Savory Snacks	8	6 (75%)	0 (0%)	1 (12%)	1 (12%)
Pantry^d^	8	5 (63%)	2 (25%)	1 (12%)	0 (0%)
Ingestible—Beverages					
Beverages	93	58 (62%)	24 (26%)	2 (2%)	9 (10%)
Ingestible—Oils					
Oil-Drops	40	27 (68%)	4 (10%)	4 (10%)	5 (12%)
Oils-Spray	11	3 (27%)	4 (36%)	2 (18%)	2 (18%)
Ingestible—Capsules					
Capsules	49	26 (53%)	13 (27%)	5 (10%)	5 (10%)
Ingestible—Sublingual Strips					
Sublingual Strips	4	0 (0%)	3 (75%)	0 (0%)	1 (25%)
Topicals					
Topicals	41	17 (41%)	9 (22%)	4 (10%)	11 (27%)
Others					
Seeds	17	1 (6%)	6 (35%)	5 (29%)	5 (29%)
Bath & Shower	13	7 (54%)	3 (23%)	2 (15%)	1 (8%)

^a^Vaping products compatible with standard 510 batteries

^b^Vaping products designed exclusively for the PAX Era vaporizer

^c^Brand-specific vaping systems where the device and pods are exclusive to a single manufacturer.”

^d^Cannabis infused edible cooking and baking ingredients and staples

### THC and CBD potency

The vast majority of inhaled products within each sub-category were categorized as very strong products, i.e., THC levels greater than or equal to 20% per gram of product (see Table 2). Ninety-seven percent (97%) of dried flower products, 96% of disposable vapes, 99% of 510 thread cartridges and infused flower, and all pax pods, closed loop pods, hash, shatter, rosin, wax, kief & sift and resin were very strong products. The majority of pre-rolls (83%) were also categorized as very strong products, although this was the lowest proportion of strong products compared to other inhaled product sub-categories.

CBD levels, however, were relatively low for most groups, with only 21% of dried flower and 20% of pre-rolls having CBD content ≥ 5%. Most vape and concentrate sub-categories had a higher proportion of products with very strong CBD levels.

No ingestible products had THC levels ≥ 20% THC or CBD levels ≥ 5% THC. All edibles were within the legal limit of a maximum of 10 mg of THC per container. Similarly, ingestible extracts were within the limit of 10 mg THC per unit (as for capsules) or per dispensed amount (as for sprays and drops) or 1000 mg THC per package (Government of Canada 2019). A lower average THC was observed for all edibles (9 mg/unit), beverages (8 mg/unit), capsules (5 mg/unit), and oils-drops (13 mg/g) compared to inhaled products. A lower or corresponding CBD level was observed for most ingestible products except for gummies (42 mg/g), oils-drops (22 mg/g), and capsules (19 mg/unit).

### Price

The average selling price of cannabis products was highest for vaping products: $159.87 ($83.16—$251.6) for disposable vapes, $153.72 ($36.5 -$240) for 510 thread cartridges, $240 for pax pods, and $144.72 ($115.9—$196.22) for closed loop pods, owing to the availability of larger cannabis sizes in grams (see Table 4). Concentrate selling prices ranged from $28.02 ($9.5—$44.94) for infused flower to $42.39 ($31.95—$49.95) for resin. The average selling price of most edible products was less than $10 except for ingestible oils; drops were sold on average for $40.74 ($14.55—$88.30) and sprays for $27.87 ($9.95—$49.90) per unit. Topicals and bath & shower products had an average selling price of $44.67 ($22.60—$68.15) and $26.37 ($13.95—$39.95) respectively.

**Table 4:** Average prices by product type (first quarter of 2024)

Category	No. of Products	M *(SD)* [Range]	
		**Price**	**Selling Price**
Inhaled—smoking (%/g)		$/g	$
Dried flower	373	7.91 (2.90) [3.57–16.54]	N/A
Pre-rolls	338	9.65 (3.62) [4.17—25.36]	23.42 (3.61) [2.98—152]
Inhaled—vaping (%/g)		$/g	$
Disposable Vapes	56	51.9 (13.7) [35—87.4]	159.87 (32.78) [83.16–251.6]
510 Thread Cartridges^a^	224	37.7 (6.2) [30—69.9]	153.72 (26.14) [36.5—240]
Pax Pods^b^	8	58.1 (5.3) [45–60]	240 (0) [240—240]
Closed Loop Pods^c^	8	36.1 (6.2) [30.5–43.8]	144.72 (35.26) [115.9–196.22]
Inhaled—Concentrates (%/g)		$/g	$
Infused Flower	143	18.66 (3.69) [10.97–28.3]	28.02 (7.55) [9.5–44.94]
Hash	44	18.99 (8.85) [10.8–49.95]	33.28 (6.27) 19.35–49.95
Shatter	21	29.38 (8.15) [15.95–40.40]	32.61 (5.58) [15.95–40.40]
Rosin	18	36.24 (17.63) [19.95–72.65]	39.82 (16.63) 12.95–72.65
Wax	3	21.36 (10.47) [15.32–33.45]	35.46 (1.74) [33.45–36.46]
Kief & Sift	6	18.59 (3.47) [15.2–23.5]	28.42 (8.62) [21.25–39.9]
Resin	29	42.39 (4.98) [31.95–49.95]	42.39 (4.98) [31.95–49.95]
Ingestible – Edibles (mg/unit, mg)		$/unit	$/package
Chocolates	20	5.49 (1.77) [2.88–9.55]	5.77 (1.53) [3.95–9.55]
Baked Goods	20	4.10 (1.27) [1.59–5.95]	4.91 (1.50) [2.7–7.95]
Gummies	130	2.32 (1.26) [0.53–8.35]	7.89 (6.76) [3.4—40]
Hard Edibles	2	N/A	6.58 (1.17) [5.75–7.4]
Savory Snacks	8	N/A	8.56 (2.21) [6.95–13.55]
Pantry (mg)^d^	8	8.38 (4.63) [2.7—15]	9.78 (4.72) [2.7–15]
Ingestible – Beverages (mg/unit, mg)		$/ml	$
Beverages	93	0.14 (0.59) [0.01—4]	7.68 (3.54) [3.50–25.65]
Ingestible – Oils (mg/g, mg/spray)		$/ml	
Oil-Drops	40	1.55 (1.16) [0.48–8.13]	40.74 (13.02) [14.55–88.30]
Oils-Spray	11	1.87 (1.51) [0.26–4.95]	27.87 (13.83) [9.95 −49.90]
Ingestible – Capsules (mg/unit, mg)		$/unit	$
Capsules	49	1.50 (0.81) [0.56 −5.9]	31.21 (13.04) [7.1–64.35]
Ingestible—Sublingual Strips (mg/unit, mg)		$/strip	$/pack
Sublingual Strips	4	2.62 (0.25) [2.5–2.99]	23.7 (6.29) [14.95 −29.95]
Topicals (mg)			
Topicals	41	N/A	44.67 (12.51) [22.60–68.15]
Others		$/unit	$
Seeds	17	N/A	41.99 (13.74) [19.6–61.9]
Bath & Shower (mg)	13	N/A	26.37 (11.81) [13.95–39.95]

^a^Vaping products compatible with standard 510 batteries

^b^Vaping products designed exclusively for the PAX Era vaporizer

^c^Brand-specific vaping systems where the device and pods are exclusive to a single manufacturer

^d^Cannabis infused edible cooking and baking ingredients and staples

The average price per unit of cannabis for the most numerous sub-categories was $7.91/g ($3.57/g—$16.54/g) for dried flower, $37.7/g ($30.0/g—$69.9/g) for 510 thread cartridges, $18.66/g ($10.97/g—$28.3/g) for infused flower, $2.32/unit for gummies, $1.55/ml ($0.48/ml–$8.13/ml) for drops, and $1.50/unit ($0.56/unit – $5.9/unit) for capsules.

A variety of quantities were available for most products in the inhaled- smoking category resulting in multiple prices for the same product although as noted in the methods section, we report single products as a range of prices and compute averages.

### 2022 vs 2024

Overall, the number of available products across all categories decreased from 2022 to 2024 (1,771 products in 2022 and 1727 products in 2024). However, changes in available product quantities varied from product to product. While the number of dried flower (373 vs 509), 510 thread cartridges (224 vs 230), pax pods (8 vs 21), beverages (93 vs 106), and chocolates (20 vs 46) available decreased in 2024 versus 2022, products such as pre-rolls (338 vs 305), disposable vapes/pens (56 vs 16), and soft chews/gummies 130 vs 104) increased. The majority of product prices per gram within sub-categories was lower in 2024 than in 2022. Average lowest dried flower prices in $/g decreased from $9.09 to $7.80 (t (857.624) = 6.031, *p* < 0.001), pre-rolls- $11.72 to $9.59 (t (641) = 7.051, *p* < 0.001), thread cartridges- $57.0 to $37.7 (t (282.738) = 15.429, *p* < 0.001) and pax pods- $89.4 to $58.1(t (25.992) = 6.926, *p* < 0.001). Average highest prices for dried flower, pre-rolls, thread cartridges and pax pods similarly decreased from $9.51 to $8.01(t (879) = 7.113, *p* < 0.001), $11.99 to $9.71(t (640) = 7.597, *p* < 0.001), $58.7 to $37.7(t (281.5) = 16.311, *p* < 0.001) and $89.4 to $58.1(t (25.992) = 6.926, *p* < 0.001) respectively. The overall average prices also decreased for disposable vapes: $120.6 to $51.9 (t (15.774) = 5.925, *p* < 0.001) and soft chews: $10.0 to $7.89 (t (232) = 2.225, *p* = 0.027) (See Table 5).

**Table 5:** Comparisons between first quarter of 2022 and 2024 by number of products, lowest price ($/g) and highest price ($/g) per product type

Product		No. of Products		Lowest Price ($/g)		Highest Price ($/g)	
		**2022**	**2024**	**2022**	**2024**	**2022**	**2024**
Flower	Dried Flower	508	373	9.09 ± 3.42	7.80 ± 2.93*	9.51 ± 3.25	8.01 ± 2.86*
	Pre-Rolls	305	338	11.72 ± 4.02	9.59 ± 3.62*	11.99 ± 4.00	9.71 ± 3.61*
				**Lowest Price ($/g)**		**Highest Price ($/g)**	
Vapes	Thread Cartridges^a^	230	224	57.0 ± 17.9	37.7 ± 6.1*	58.7 ± 18.5	37.7 ± 6.2*
	Pax Pods^b^	21	8	89.4 ± 18.8	58.1 ± 5.3*	89.4 ± 18.8	58.1 ± 5.3*
				**Average Price ($/g)**			
	Disposable Vapes	16	56	120.6 ± 45.8	51.9 ± 13.7*		
				**Selling Price**			
Edibles	Soft Chews	104	130	10.0 ± 7.72	7.89 ± 6.76*		
	Beverages	106	93	8.14 ± 4.47	7.68 ± 3.54		
	Chocolates	46	20	5.50 ± 1.49	5.77 + 1.53		

^*^ *p*-value < 0.05

^a^Vaping products compatible with standard 510 batteries

^b^Vaping products designed exclusively for the PAX Era vaporizer

The low and high THC potencies (%/g) of most products were significantly higher in 2024 in contrast with the data provided in 2022; dried flower: 23 vs 19 (t (656.957) = −13.582, *p* < 0.001), pre-rolls: 22 vs 18) (t (610.381) = −9.984, *p* < 0.001), 510 thread cartridges: 79 vs 71 (t (386.985) = −5.722, *p* < 0.001), pax pods: 81 vs 71 (t (24.081) = −3.802, *p* < 0.001), disposable vapes: 79 vs 61 (t (17.457) = −2.352, *p* = 0.031) and beverages: 8 vs 5 (t (196.617) = −5.640, *p* < 0.001) (See Table 6).

**Table 6:** Tetrahydrocannabinol (THC) and cannabidiol (CBD) potency by product type in first quarter of 2022 vs 2024

Product		Low THC (%/g)		High THC (%/g)		Low CBD (%/g)		High CBD (%/g)	
		**2022**	**2024**	**2022**	**2024**	**2022**	**2024**	**2022**	**2024**
Flower	Dried Flower	18.99 ± 3.70	23.13 ± 4.97*	24.77 ± 4.09	29.38 ± 5.37*	0.27 ± 1.57	0.32 ± 1.80	1.77 ± 2.45	2.60 ± 3.23*
	Pre-Rolls	17.98 ± 3.66	21.46 ± 5.11*	23.96 ± 3.92	27.71 ± 5.38*	0.26 ± 1.37	0.36 ± 1.79	1.64 ± 2.38	2.64 ± 3.50*
Vapes	Thread Cartridges^a^	70.99 ± 18.31	79.18 ± 11.51*	78.10 ± 18.27	84.77 ± 11.52*	4.60 ± 14.82	1.35 ± 7.22*	6.83 ± 16.54	5.44 ± 9.13
	Pax Pods^b^	70.69 ± 11.60	80.88 ± 2.47*	76.57 ± 12.01	86.88 ± 2.47*	4.35 ± 11.77	0.00 ± 0.00	6.53 ± 12.76	0.13 ± 0.35*
	Disposable Vapes	61.73 + 28.32	79.04 ± 14.96*	67.56 ± 30.33	84.95 ± 15.21*	16.50 ± 25.56	2.71 ± 12.26	18.95 ± 28.6	5.99 ± 13.42
		**Low THC (mg)**		**High THC (mg)**		**Low CBD (mg)**		**High CBD (mg)**	
Edibles	Soft Chews	8.67 ± 3.26	9.14 ± 2.56	8.69 ± 3.24	9.32 ± 2.39	49.12 ± 140.11	41.34 ± 139.82	49.22 ± 140.08	43.52 ± 148.29
	Beverages	4.92 ± 4.03	7.89 ± 3.38*	4.94 ± 4.01	8.25 ± 3.33*	9.81 ± 16.43	7.93 ± 18.62	9.94 ± 16.41	8.41 ± 18.85
	Chocolates	9.26 ± 2.50	9.93 ± 0.34	9.33 ± 2.26	10.00 ± 0.00	3.39 ± 7.88	2.75 ± 4.33	3.52 ± 7.83	2.95 ± 4.22

^*^ *p*-value < 0.05

^a^Vaping products compatible with standard 510 batteries

^b^Vaping products designed exclusively for the PAX Era vaporizer

CBD potencies, however, decreased for these same products except for flower products which saw a more significant increase in average high CBD potency for both dried flower: 2 vs 3 (t (666.437) = −4.154, *p* < 0.001) and pre-rolls: 2 vs 3 (t (597.687) = −4.280, *p* < 0.001). There were no significant changes in CBD potencies of edibles.

## Discussion

The purpose of the present study was to catalog all cannabis products available to consumers in the Ontario cannabis legal market in the first quarter of 2024, five years into legalization and two years since our last review of available market offerings. In our study, we found an increase in the number of non-flower cannabis products such as vapes and edibles, although inhaled products remained the most abundant products on the market. The prices of flower products- dried flower and pre rolls, some vape products and soft chews significantly declined. THC potency of products available to consumers increased for all flower products, the most abundant vape products (disposable vapes and thread cartridges) and beverages, while CBD potencies remained low for most products, decreased for pax pods and increased for flower products. With regards to marketed plant types, flower and concentrates were predominantly indica, most vapes were sativa-dominant while edibles and other product types were likely to be a blend or hybrid.

Similar to our data from our 2022 survey of the OCS (Tassone et al. 2023), inhaled products still dominated, accounting for about 71% of all products (equaling the ≈71% observed in 2022). Although smoking continues to be the most common route of administration (Hammond et al. 2022; Goodman et al. 2020; Health Canada 2024), the increase in the proportion of edible products, particularly gummies, in the market correlates with the increasing preference for edibles consumption observed in the Canadian population and in other legal markets (McDonald et al. 2025; Goodman et al. 2020; Rotermann 2021; 2024; 2018; Goodman et al. 2024). Likewise, the number of vapes available in the market tripled, while pre-rolls marginally increased. Growing preference for edibles may be influenced by factors such as the discretion associated with edible use, a desire to avoid exposure to combustion by-products, and longer time course of effects (Rotermann 2021; Donnan et al. 2024) and lower-risk cannabis use guidelines recommend the use of other routes of administration besides smoking (Fischer et al. 2022). This may also be driven by the resemblance of edible forms such as gummies to common edible items such as snacks or candy (Han and Shi 2025). Novel cannabis consumers may also opt for more familiar ingestible forms, for example, ingesting a gummy which is similar to usual habits such as eating or taking medication, versus acquiring new habits such as smoking a substance. However, due to the delayed onset of effects, the consumption of edible cannabis products has been associated with increased risk of health harms, reports of accidental pediatric ingestions, and increased cannabis-related emergency room visits and hospitalizations (Hammond et al. 2022; Dilley et al. 2023; Rotermann 2021; Donnan et al. 2024). Effective outreach is needed to educate users about the potential risks of some of these newer methods of use.

There are also implications of the change in pricing of cannabis products. The average price per unit of dried cannabis and edibles, particularly gummies or soft chews significantly decreased between 2022 and 2024. A similar continuous decline in retail cannabis prices in Washington, Colorado, and Oregon post- legalization has been observed (Freeman et al. 2019; Smart et al. 2017**).** Self-reported prices of cannabis products such as flowers, edibles, drinks, and concentrates also indicate a narrowing of price differences between illegal and legal markets, with the exception of vapes which are still priced significantly lower in the illegal market (Rundle et al. 2025). Studies have shown that young adults and people who use cannabis daily are price sensitive (Ben Lakhdar et al. 2016; Chiu et al. 2021). Price control strategies have been determined to be an effective approach in decreasing negative impact of cannabis on public health and preventing excessive cannabis consumption (Dilley et al. 2023; Han et al. 2025). Historically, tobacco price control has been crucial in influencing tobacco consumption, particularly among the youth (Hammond et al. 2020). Decades of alcohol research has also identified marketing, pricing, and availability as key determinants for drug use and health harms (Manthey et al. 2023). Strategies such as taxation and minimum unit pricing for alcohol have been proven to be effective in reducing consumption (Wagenaar et al. 2009; Holmes et al. 2014). Cannabis price control is therefore an important determinant in consumer purchasing behavior, consumption, and the market shift from illegal to legal cannabis markets, a vital aim of legalization (Hammond et al. 2020; Dodd et al. 2025; Dilley et al. 2023). As noted in our previous study (Tassone et al. 2023), the continuous decline of legal cannabis prices will facilitate legal sourcing of cannabis, as higher prices are a key deterrent for legal cannabis purchase (Goodman et al. 2022). This is consistent with reports that more consumers are purchasing from legal sources whereas purchasing from all other sources including the illegal market has declined compared to 2018 (Health Canada 2024; Health Canada 2018; Rundle et al. 2025).

In the present synthesis, a shift toward more high potency products was observed, a development that has been regarded as a public health concern (Goodman et al. 2020; Mahamad et al. 2020). The average potency of high-THC dried flower products in Ontario has further increased since 2022 from 24.7% to 29.4%, ≈19% (5 percentage points) increase in potency in two years. High-potency products can have a negative impact on mental health and addiction outcomes and have been associated with the development of dependence and cannabis use disorder (Freeman et al. 2019; Hammond et al. 2022; Lake et al. 2025; Marinello 2025). With increasing accessibility to high-THC products, misconceptions regarding cannabis safety and altering perceptions of harm, particularly the downplay of side-effects and risk, could result (Pennypacker et al. 2022). The development of specific policy measures to regulate the legal cannabis market is therefore key (Goodman et al. 2020). Restrictions on THC potency, as implemented in Uruguay (Marzetti 2025), and an introduction of a potency- based tax, where taxes are applied based on THC content, have been proposed in curbing the consumption of high-potency products and reducing harm (Kilmer and Perez-Davila 2023; Hall and Lynskey 2020). Currently, the impact of THC potency restrictions on consumer purchase decisions is unclear. Dodd et al., 2025 (Dodd et al. 2025), however, raise a concern that introducing higher taxes may challenge the displacement of the illicit market by the legalized market (Donnan et al. 2024). Governmental restrictions on THC potency in Uruguay (no more than 15% THC per flower product) and similar strict legal efforts in Quebec (30% weight per weight THC per flower product, 10 mg/package for solid edibles (maximum of 5 mg per discrete unit) and maximum 5 mg/container for liquid edible cannabis products) may be contributing to consumer preference for products from illegal markets despite legalization (Queirolo 2024; Wadsworth et al. 2023). Donnan et al., 2024 (Donnan et al. 2024), found that about a third of consumers in Canada, driven by higher THC potencies currently unavailable in legal cannabis markets, were likely to purchase cannabis edibles from illegal sources (Donnan et al. 2024).

CBD potency remained lower compared to THC potency across all products. Compared to 2022 (Tassone et al. 2023), with the exception of dried flower and pre-rolls whose CBD levels increased marginally, all other products had comparable or lower CBD potency. The association between THC:CBD ratio and cannabis associated harm remains unclear. Some studies have suggested CBD may offset THC related harm. CBD has been proposed to decrease the negative psychotropic effects and potentiate positive therapeutic effects of THC (Navarrete 2021). CBD has also been suggested to play a potential role in cannabis harm reduction and treatment of other substance use disorders such as nicotine and opioid use disorder in human studies (Paulus et al. 2022). Self-report from people who use cannabis have also corroborated a reduction in cannabis use and withdrawal symptoms with use of CBD-rich products (Fortin et al. 2022). Recent laboratory research has however, suggested that CBD may not meaningfully impact the effects of THC (Englund et al. 2023), with one trial finding that CBD actually exacerbated the acute negative effects of THC on verbal memory and psychotic symptoms in patient with schizophrenia (Chesney et al. 2025). The finding that both THC and CBD content increased (marginally for the latter) in dried flower and pre-rolls is therefore difficult to interpret. THC is psychoactive, while CBD is not, although both can have medicinal properties. A study by Shi, Cao (Shi et al. 2019) found that regardless of the type of respondent- never users, former users, recreational and medical users or users of both-, higher CBD products were more likely to be chosen. A finding which the authors attributed to possible user considerations of positive therapeutic effects and no intoxication associated with CBD. This may suggest an increasing preference for products with higher CBD potency and may explain the marginal increase observed.

The majority of inhaled products available were indica-dominant and had very strong THC levels. Conversely, vape products were predominantly sativa-dominant whereas most ingestible products were a blend or hybrid. Indica-dominant products have been associated with lower subjective ratings of arousal, generating a feeling of ‘relaxation’ and ‘sleepiness’. Conversely, sativa plant types are preferred for euphoria and increased energy levels (Pearce et al. 2014; Sholler et al. 2022; Okey et al. 2023). Consumers reported preference for indica in the evenings while sativa would be preferred in the daytime or at a party indicating plant type preferences may be influenced by situational factors (Sholler et al. 2022), a finding reflected by the varying plant type availability in the market to meet varying consumer preferences. However, the utility of the distinction between sativa and indica effects is still debated. A study by Stith et al., 2019 (Stith et al. 2019), found that only THC potency levels was an independent determinant of subjective effects once other characteristics of cannabis were controlled for. Higher THC potency was associated with greater effects regardless of plant type. The relevance to health outcomes and policy implications of these classifications remains unclear. These terms are mainly commercial designations utilized in marketing contexts to appeal to consumers but provide no evidence-based insight on product psychoactive effects which are more accurately determined by laboratory analysis of cannabinoid and terpenoid profiles (Lewis et al. 2018; Smith et al. 2022).

Some limitations should be considered in the interpretation of our findings. This study outlines products available in the legal Ontario market and does not infer what consumers are purchasing overall; although purchasing trends may likely be influencing changes in the market between 2022 and 2024. These data do not also take into consideration, illegal sources of cannabis available to consumers in Ontario. The scope of this study is limited to online retail data on the OCS website and does not capture products from physical retail locations. This may limit the generalizability of findings although OCS being the sole distributor in Ontario curbs this to an extent. We also utilize averages of price and potency which may be impacted by outliers or extreme values. The selling prices presented were not useful for data comparisons and interpretation but may provide insight on real-world consumer contexts. Product prices in different sub-categories on the OCS website were expressed in different units which limited price comparisons across sub-categories.

## Conclusion

The Ontario cannabis market is evidently rapidly evolving, indicating the need for continuous surveillance of vital market determinants such as availability, product type, potency, and price that influence economic and public health consequences. This is a requisite in assessing the impact of cannabis legalization and harms. The wide availability of high-THC potency cannabis products coupled with decreasing prices could threaten public health, which warrants further surveillance of market trends. Stricter regulations on product potencies should be considered to regulate potency levels and thereby manage the availability of high-potency products, which may be associated with increased problematic use. Price management strategies such as potency- based taxing, tiered offerings and minimum unit pricing that will balance market prices to prevent overconsumption and concurrently maintain preference for legal markets should also be considered.

What does this study add to existing knowledge? This study provides evidence that the price of legal cannabis has dropped over time, while the potency has increased.

What are the key implications for public heath interventions, practice and policy? Given that the purchasing choices of consumers is based partly on price, changes in price and potency over time have implications for the safety of products available on the legal market.

## Data Availability

The corresponding author will consider requests for the data.
